# Translation of microwave methodology to continuous flow for the efficient synthesis of diaryl ethers via a base-mediated S_N_Ar reaction

**DOI:** 10.3762/bjoc.7.160

**Published:** 2011-10-04

**Authors:** Charlotte Wiles, Paul Watts

**Affiliations:** 1 Chemtrix BV, Burgemeester Lemmensstraat 358, 6163 JT, Geleen, The Netherlands; 2 Department of Chemistry, The University of Hull, Cottingham Road, Hull, HU6 7RX, UK

**Keywords:** automated synthesis, continuous flow, microreactor, microwave, nucleophilic substitution, organic bases

## Abstract

Whilst microwave heating has been widely demonstrated as a synthetically useful tool for rapid reaction screening, a microwave-absorbing solvent is often required in order to achieve efficient reactant heating. In comparison, microreactors can be readily heated and pressurised in order to “super-heat” the reaction mixture, meaning that microwave-transparent solvents can also be employed. To demonstrate the advantages associated with microreaction technology a series of S_N_Ar reactions were performed under continuous flow by following previously developed microwave protocols as a starting point for the investigation. By this approach, an automated microreaction platform (Labtrix^®^ S1) was employed for the continuous flow synthesis of diaryl ethers at 195 °C and 25 bar, affording a reduction in reaction time from tens of minutes to 60 s when compared with a stopped-flow microwave reactor.

## Introduction

**Figure 1:**
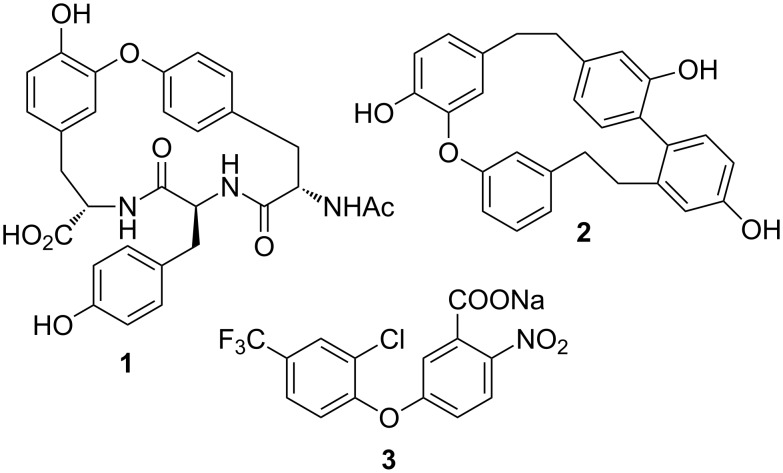
Illustration of synthetically interesting diaryl ethers.

Diaryl ethers are a synthetically interesting subunit [1], with examples found in a series of medicinally significant natural products, such as (−)-K-13 (**1**) [2], riccardin C (**2**) [3] and combretastatins [4], along with synthetic herbicides, such as RH6201 (**3**) [5] (Figure 1). Installation of the diaryl ether can, however, be synthetically challenging, and this is illustrated by the wide number of techniques developed, which include Ullmann ether synthesis [6], Pummerer-type rearrangements [7], Buchwald–Hartwig couplings [8], phenolic additions to amines [9], fluoride mediated couplings [10–11], and the use of solid supports [12].

**Scheme 1:**
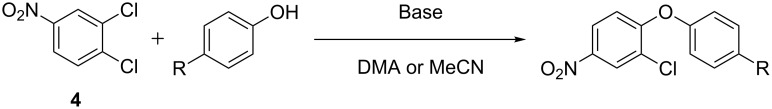
Illustration of the model reaction used to compare the enabling technologies of microwave and microreactor synthesis.

Until recently, the nucleophilic substitution of aromatic halides to phenolic substrates has been largely overlooked, with Ueno and coworkers [13] reporting the use of triethylsilane and a phosphazene “super base”, Holmes [14] describing the use of scCO_2_, and Moseley et al. [15] employing microwave irradiation as a means of efficiently heating the reaction mixture in order to significantly reduce reaction times (Scheme 1). Although microwaves have found widespread application in the research laboratory, their implementation at a large scale, whilst increasing, is not as well established, largely due to the challenges associated with the uniform irradiation of large reactor vessels [16].

In a critical assessment of microwave-assisted organic synthesis, Moseley and Kappe [17] recently concluded that on a small scale (1 to 50 mL) any energy savings made as a result of using microwave irradiation were attributable to the reduction in reaction time achieved through the use of sealed vessels, and not because microwave irradition is a more energy efficient method of heating. When considering large-scale reactors [18], multimode microwave reactors have been found to be more energy efficient than small single-mode systems, but not more efficient than conventional heating, due to their minimal penetration depth [19]. Coupled with the fact that microwave heating is eight times more expensive than conventional heating [20], techniques for efficient heat transfer are required if costs are to be reduced, particularly at the production level.

Looking towards another emerging technology, that of continuous-flow methodology, Kappe and coworkers [21] and Ryu et al. [22] demonstrated that the “microwave effect” can be mimicked in high-temperature flow reactors, which can be scaled to increase production volume without changing the reaction conditions employed [23–25], resulting in a reduction in energy usage per mole. With this in mind, we report herein the translation, and further development, of a microwave method for the S_N_Ar reaction of chloroarenes to a series of *para-*substituted phenols to afford a general and efficient route to the diaryl ether subunit.

## Results and Discussion

**Scheme 2:**
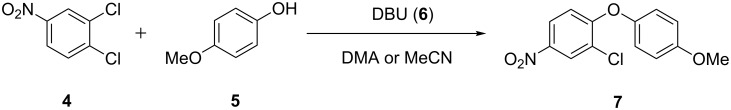
Illustration of the model reaction used to benchmark Labtrix S1 against batch and stopped-flow microwave reactors.

With the optimised conditions from Marafie and Moseley’s [15] stopped-flow investigation taken as a starting point, the synthesis of diaryl ethers (Scheme 2) was investigated under continuous-flow conditions. As the continuous-flow reactor enables the reaction chamber to be maintained at the reaction temperature (once the steady state is reached) time is not wasted for heating and cooling of the stopped-flow “batches”. Consequently, the system has the potential to be more efficient. The quantity of material generated can therefore be determined by the length of continuous operation and not by the number of “batches” performed.

**Figure 2:**
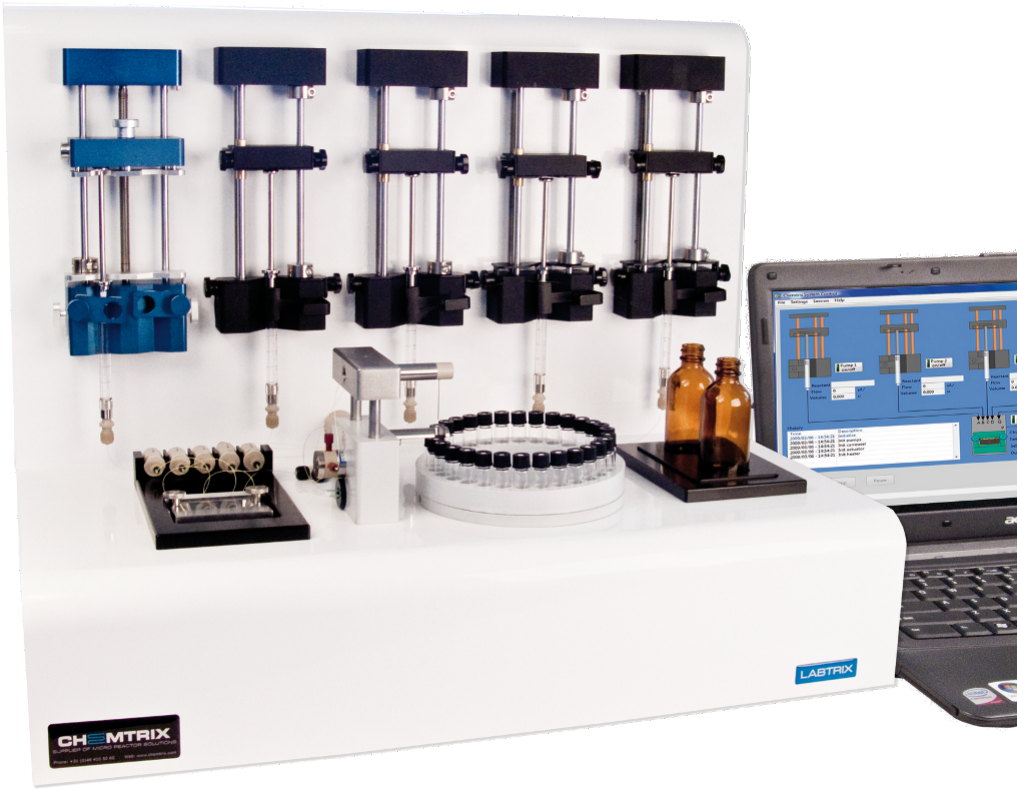
Photograph illustrating Labtrix^®^ S1, the automated microreactor development apparatus from Chemtrix BV (NL), used for the evaluation described herein.

To perform the flow reactions, the microreactor development apparatus Labtrix^®^ S1 (Chemtrix BV, NL), illustrated in Figure 2, was employed. The heart of the system is a glass microreactor that is positioned on a thermally regulated stage, which enables reactions to be performed between −15 and 195 °C. Reagent solutions are delivered to the reactor through a series of syringe pumps (0.1 to 25 µL·min^−1^) and the system is maintained under a back pressure of 25 bar, which enables reactants and solvents to be heated above their atmospheric boiling point whilst staying in the liquid phase. The reactant flow rates, reactor temperature and sample collection point is automated and the system has an in-line pressure sensor that monitors the system pressure throughout the course of an investigation. The software enables the effect of reaction time, temperature and reactant stoichiometry to be investigated in an automated manner whilst the system is operated, unattended, within a fume cupboard.

**Figure 3:**
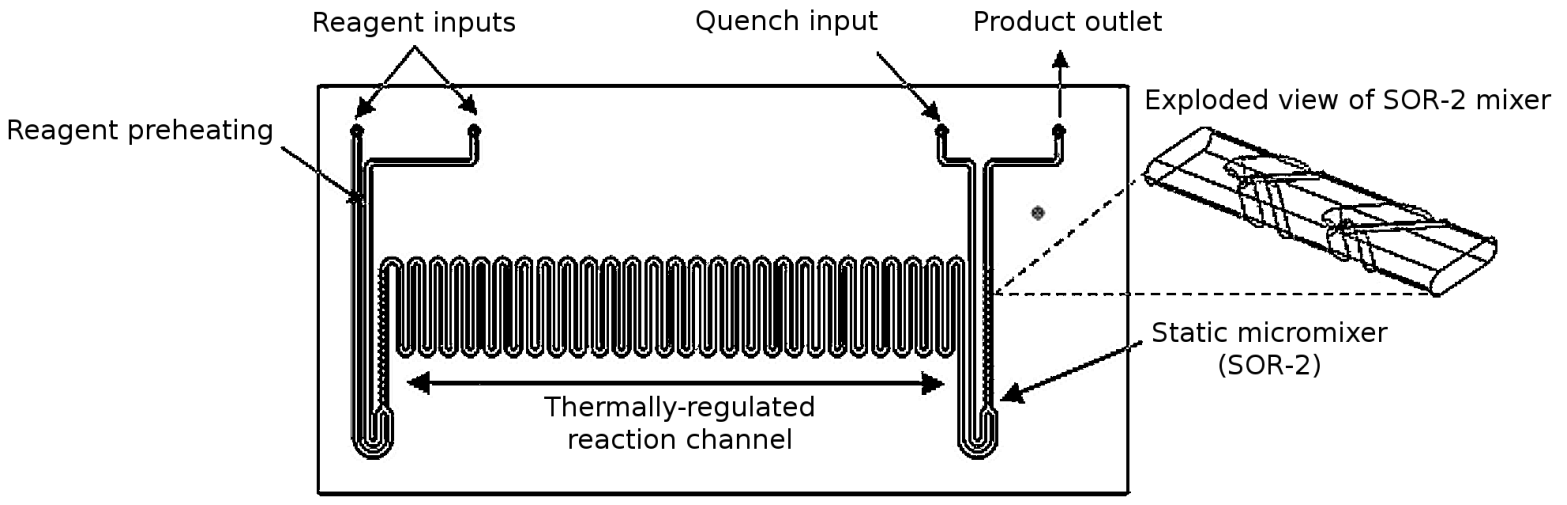
Schematic illustrating the 10 µL reactor manifold used for the S_N_Ar reactions described herein (3223; Chemtrix BV, NL), with the important features highlighted.

The glass microreactors employed herein have a footprint of 44 mm × 22 mm and contain etched microfluidic channels (300 µm (wide) × 120 µm (deep)) in which the reactions take place. By varying the channel length a series of reactor volumes can be accessed (3221 (1 µL), 3222 (5 µL), 3223 (10 µL) and 3227 (19.5 µL)). In order to increase the efficiency of thermal and mass transport on the microscale, the devices contain preheating channels, which bring reagents to the reaction temperature ahead of mixing, and static micromixers (staggered oriented ridge (SOR-2)) [26] are incorporated where any two reagent streams meet in order to increase the efficiency of mixing (*T*_mix_ ≤ 0.3 s), compared with T-mixers, and to increase the remaining channel volume available for reaction (Figure 3).

**Figure 4:**
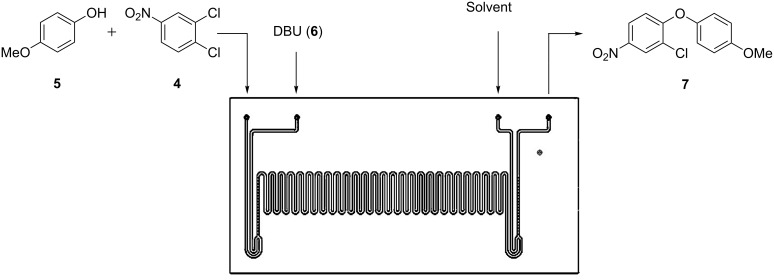
Schematic illustration of the reactor manifold used to evaluate the continuous-flow synthesis of 2-chloro-1-(4-methoxyphenoxy)-4-nitrobenzene (**7**) in the presence of DBU (**6**).

**Figure 5:**
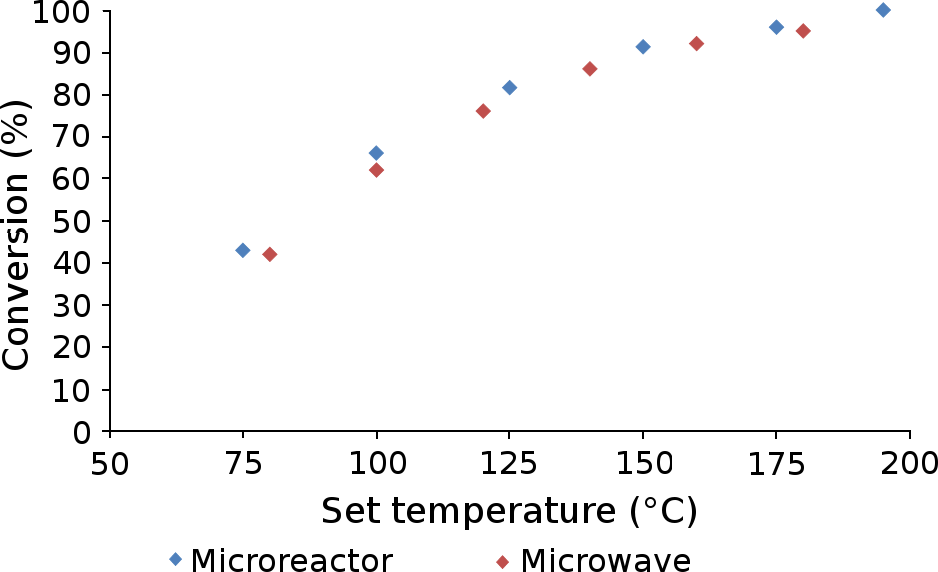
Comparison of the results obtained in Labtrix^®^ S1 with reported data generated in a microwave synthesiser.

Employing a two-feed system, Figure 4, where one stock solution contained 3,4-dichloronitrobenzene (**4**, DCNB) and 4-methoxyphenol (**5**, 1.3 M and 1.56 M respectively) in dimethylacetamide (DMA) and the second 1,8-diazabicycloundec-7-ene (DBU, **6**, 1.95 M) in DMA, we investigated the nucleophilic substitution reaction under flow conditions. By using a reaction time of 10 min, achieved by setting a total flow rate of 1 µL·min^−1^, the effect of reactor temperature on the synthesis of 2-chloro-1-(4-methoxyphenoxy)-4-nitrobenzene (**7**) was investigated. Reactions were initially performed in the absence of a base, in order to monitor the background reaction by means of offline GC-FID analysis. After a reaction time of 10 min at a reactant temperature of 195 °C, analysis of the reaction products by GC-FID confirmed no background reaction had occurred, with DCNB (**4**) and 4-methoxyphenol (**5**) recovered without reaction or degradation. Introducing DBU (**6**) into the reactor produced comparable results in the glass microreactor to those reported by Moseley et al. [15] (Figure 5).

**Effect of reaction time:** Satisfied by this result, we looked at increasing the efficiency of the reaction, and thus we subsequently investigated the effect of reaction time at 195 °C with a view to increasing the space yield time. This approach was successful and revealed that the reaction did not require a 10 min reaction time, with quantitative conversion of DCNB (**4**) to the diaryl ether **7** achieved in 60 s. It is important to note that no degradation of the diaryl ether **7** was observed when extended reaction times of up to 45 min were employed. The ability to decrease the reaction time required in flow when compared to the microwave methodology can be rationalised if you think that part of the reaction time for a microwave reaction involves the heating up and cooling down of the system, and it is this increase in processing time that is removed by a flow reactor once it has reached steady state.

**Effect of reaction solvent:** When performing reactions under microwave irradiation, it is important to select a solvent that is not transparent to microwave radiation in order to ensure efficient heating of the reaction mixture. With this in mind, the method reported in the literature employed DMA, but the high boiling point of the solvent (164–166 °C) makes it difficult to isolate the diaryl ethers when reactions are performed on a small scale. Consequently, the reaction was investigated in a series of solvents with low boiling points. The microreactions were performed under 25 bar of back pressure, which means that solvents such as acetonitrile (MeCN) can be readily employed at temperatures exceeding their atmospheric boiling point (81–82 °C), and upon replication of the investigation summarised in Figure 5, comparable results were obtained, illustrating that MeCN is a suitable alternative to DMA.

**Figure 6:**
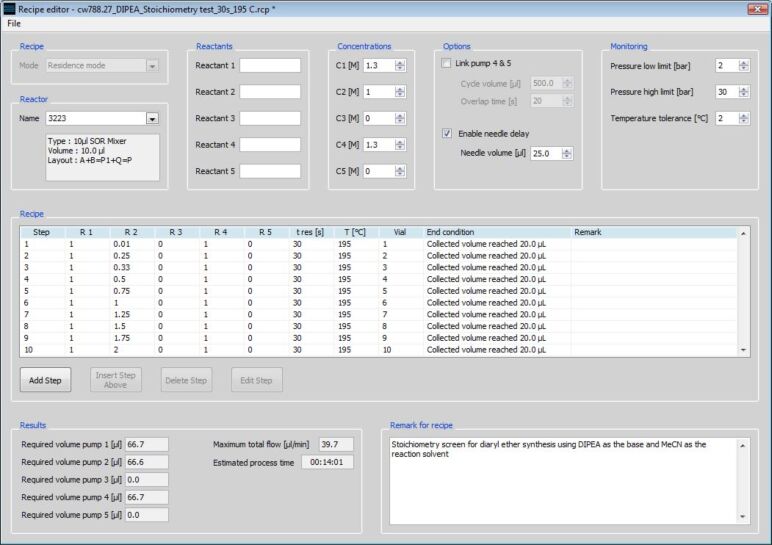
Screen shot from the Labtrix^®^ S1 control software illustrating the system file that enables the user to readily program a stoichiometry screen; this example varies the base stoichiometry at a fixed reaction time and temperature, however, the user can alter all 3 variables at any point.

**Screening of organic bases:** With one of the salient features of microreaction technology being the speed of reaction optimisation, due to the low system hold-up volume, we subsequently investigated the effect of base type and stoichiometry on the reaction in MeCN. Whilst this would conventionally be performed by preparing a series of solutions with different base concentrations, the control software for Labtrix^®^ S1 enables facile programming of reactant stoichiometries from a single stock solution. Figure 6 illustrates the use of a 1.0 M DCNB (**4**) 4-methoxyphenol (**5**) solution (reactant A), a 1.0 M base solution (reactant B) and MeCN as the diluent (added through the quench input (Figure 4)). At this stage, it was also decided that the 4-methoxyphenol (**5**) equivalents would be reduced from 1.2 to 1.0 equiv in order to reduce the post reaction purification required in order to isolate the diaryl ether **7** in high purity.

Using this approach, we investigated stoichiometries from 0.01 to 2.00 equiv for 10 additional organic bases (Table 1), with 1.0 M stock solutions prepared owing to the variable miscibility of the selected bases with the reaction solvent, MeCN. In order to gauge the effect of the base, reactions were performed under the suboptimal conditions of 30 s at 195 °C, with each screen taking only 14 min to generate the samples for analysis.

**Table 1:** Illustration of the organic bases investigated, ranked in order of increasing basicity and the conversion to diaryl ether **7** obtained when 1 equiv of base was used (residence time = 30 s; reactor temperature = 195 °C).

Entry	Organic base (1 equiv)	p*K*a	Conversion (%)

1	Pyridine	5.20	3.00
2	1,4-Diazabicyclo[2.2.2]octane (DABCO)	5.60	5.01
3	Lutidine	6.75	0.00
4	Tetramethylethylenediamine (TMEDA)	8.97	0.00
5	*N-*Methylpiperidine	10.08	1.62
6	Diisopropylethylamine (DIPEA)	10.50	25.27
7	Triethylamine	10.70	0.85
8	Tetramethylpiperidine (TMP)	11.07	0.00
9	1,8-Diazabicycloundec-7-ene (**6**, DBU)	12.00	55.54
10	1,5-Diazabicyclo(4.3.0)non-5-ene (DBN)	12.80	56.27
11	1,1,3,3-Tetramethylguanidine (TMG)	13.60	55.10

**Figure 7:**
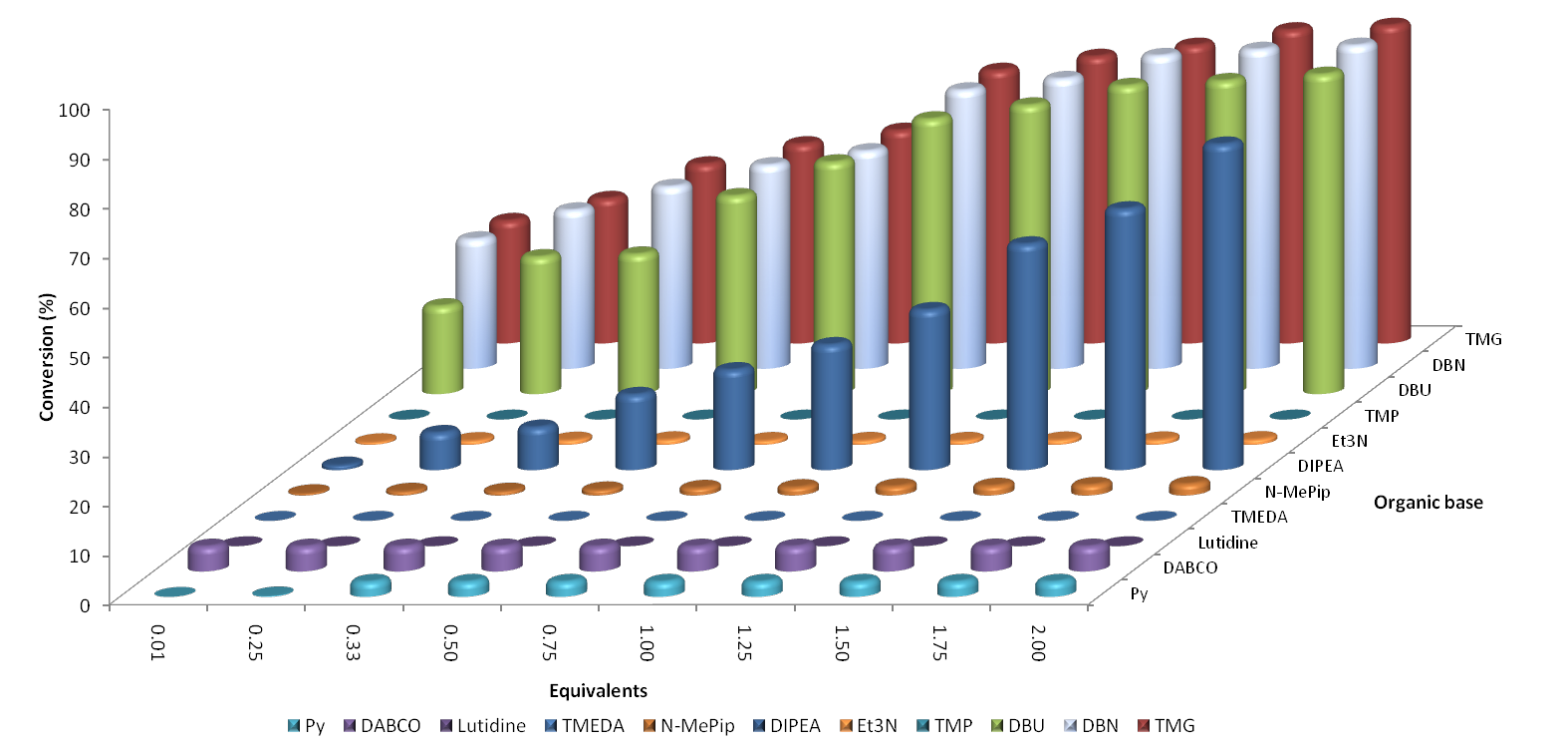
Summary of the results obtained for the organic base screen towards the S_N_Ar reaction between DCNB (**4**) and 4-methoxyphenol (**5**) (residence time = 30 s; reactor temperature = 195 °C).

As Figure 7 illustrates, a wide range of reactivities was obtained, with pyridine, *N-*methylpiperidine, tetramethylpiperidine, lutidine, tetramethylethylenediamine (TMEDA) and triethylamine affording negligible conversions of DCNB (**4**) to 2-chloro-1-(4-methoxyphenoxy)-4-nitrobenzene (**7**). In comparison, DBU (**6**), 1,5-diazabicyclo(4.3.0)non-5-ene (DBN) and 1,1,3,3-tetramethylguanidine (TMG) afforded 55.5, 56.3 and 55.1% conversions (at 1 equiv), respectively. If we compare the results obtained with the dissociation constant of the bases employed (Table 1), a clear link can be seen. Importantly, in all cases the hydrochloride salt of the base, formed as a byproduct in the reaction, remained in solution.

With this information in hand, we retained DBU (**6**) as the base (1.5 equiv) and 2-chloro-1-(4-methoxyphenoxy)-4-nitrobenzene (**7**) was synthesised in 99.7% yield with a reaction time of 60 s at 195 °C, affording a throughput of **7** of 108.5 mg·h^−1^ with a 1:1 ratio of 4-methoxyphenol (**5**) and DCNB (**4**).

**Figure 8:**
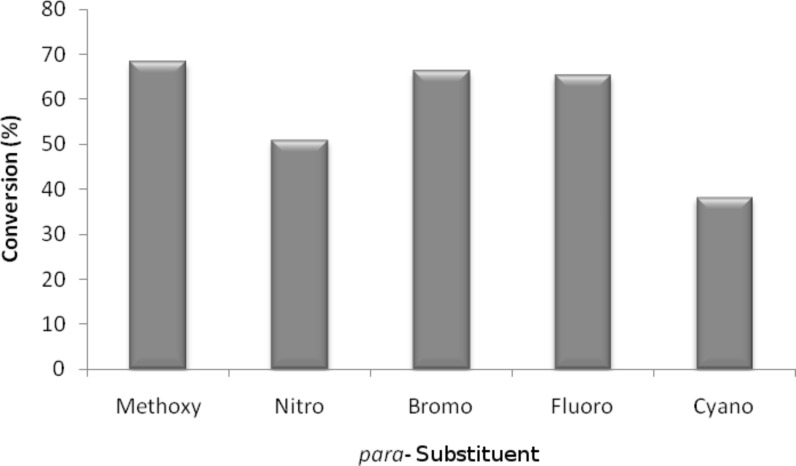
Illustration of the substituent effect on the synthesis of diaryl ethers under continuous flow (residence time = 30 s; reactor temperature = 195 °C).

**Effect of phenol substitution.** Having optimised the reaction for the synthesis of 2-chloro-1-(4-methoxyphenoxy)-4-nitrobenzene (**7**), the next step in the investigation was to evaluate the effect of the *para-*substituent on the phenolic derivative. To do this a series of commercially available phenols were evaluated; 4-nitrophenol (**8**), 4-cyanophenol (**9**), 4-bromophenol (**10**) and 4-fluorophenol (**11**). Again after a reaction time of 30 s and a reactor temperature of 195 °C, the reactivities of the four phenols were compared before each reaction was optimised for diaryl ether isolation. As expected, Figure 8 illustrates that those phenol derivatives bearing an electron-donating substituent were found to be more reactive.

For each *para-*substituted phenol, the reaction time was optimised and, as Table 2 illustrates, this enabled the synthesis of five diaryl ethers in high yield and excellent purity as verified by MS and NMR spectroscopy. Compared to the work of Moseley [15], the use of a flow reactor meant that it was possible to optimise the reaction of 4-cyanophenol (**9**) to obtain 2-chloro-1-(4-cyanophenoxy)-4-nitrobenzene (**13**) in >99% yield, compared with 42% in the microwave reactor.

**Table 2:** Summary of the reaction conditions employed for the DBU (**6**) mediated S_N_Ar reaction under continuous flow.

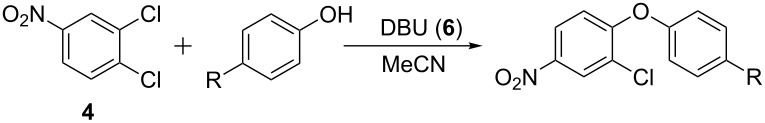				

Phenol		Residence time (min)	Conversion (%)	Yield (%)

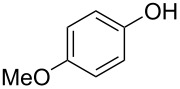	**5**	1	quant.	99.69
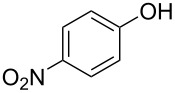	**8**	5	quant.	99.84
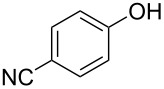	**9**	10	quant.	99.72
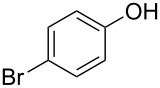	**10**	1	quant.	99.86
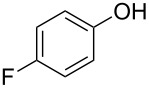	**11**	2	quant.	99.79

**Use of inorganic bases:** Whilst the use of organic bases enabled a comparison of microwave and microreactor technologies to be performed, the use of organic bases can be viewed as disadvantageous due to their relatively high cost compared with inorganic bases [27]. In addition, standard “batch” conditions afforded slurries and were identified by Moseley [15] as being disadvantageous for the stopped-flow microwave reactor. Herein, we employed an aqueous solution of K_2_CO_3_, which resulted in a biphasic microreaction system. In batch this approach would prove disadvantageous as it would result in a biphasic system in which poor mass transport between the organic and aqueous layers would reduce the reaction rate.

**Figure 9:**
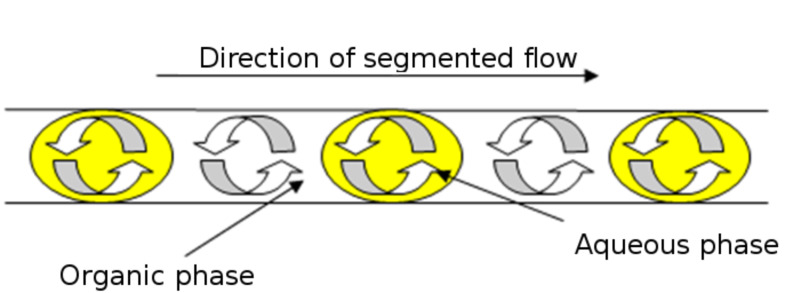
Schematic illustrating the mixing of immiscible reagent streams in a microfluidic channel, whereby the continuous phase composition depends on the ratio of organic and aqueous reactants employed.

In a microfluidic channel, reproducible droplets can be formed within a continuous phase, giving rise to a high interfacial surface area, with mixing further promoted by internal circulation within the droplets (Figure 9). With this in mind the use of aqueous K_2_CO_3_ as the base was investigated as a means of simplifying postreaction processing and reducing the costs associated with the synthetic methodology developed.

**Figure 10:**
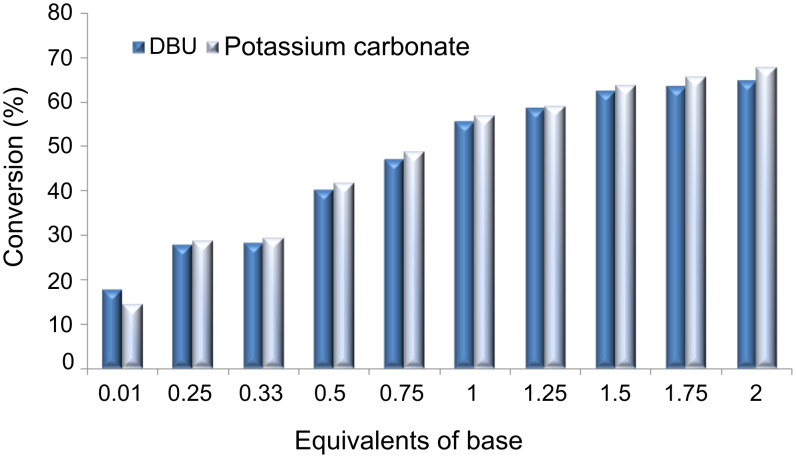
Comparison of base effect on the synthesis of 2-chloro-1-(4-methoxyphenoxy)-4-nitrobenzene (**7**) (residence time = 30 s; reactor temperature = 195 °C).

Using this approach, we investigated the effect of K_2_CO_3_ stoichiometry at 195 °C with a reaction time of 30 s, and, as Figure 10 illustrates, comparable results to those obtained for DBU (**6**) were recorded.

Under the optimal conditions, the target compound **7** would be obtained in a throughput of 109 mg·h^−1^ however this can be increased by using a more concentrated base solution and therefore reducing the proportion of the second solution within the reaction channel. Increasing the K_2_CO_3_ concentration to 4.5 M enabled the throughput to be increased to 152 mg·h^−1^ whilst maintaining a 1:1:1.5 reactants-to-base ratio.

## Conclusion

Employing a microreactor with a small hold-up volume enabled us to screen a large number of reaction conditions using only mg quantities of substrate. Using this approach, we were able to build on the methodology developed by Moseley and coworkers [15]; by replacing the high-boiling-point reaction solvent DMA with MeCN and simultaneously reducing the proportion of phenol derivative employed, product isolation was more facile. Furthermore, in the case of 4-methoxyphenol (**5**) it was possible to reduce the reaction time from tens of minutes to 60 s at a reactor temperature of 195 °C; a time saving which can be attributed to the efficient heat transfer obtained within the microreactor, and the fact that in a microwave reactor the time to heat up and cool down the system can significantly increase the reaction time.

In addition, compared to previously reported microwave methodologies, employing a microreactor enabled us to investigate the use of a biphasic reaction system, reducing the costs associated with the transformation through the use of an inorganic base, to afford the target diaryl ethers in high yield and purity, after a simple offline aqueous extraction.

Whilst it can be seen from the data described herein that microreaction technology can be utilised for the rapid generation of reaction information and small quantities of isolated materials, the production volume of such units is inherently small. With efficient heat and mass transfer key to the success of microreactors, it is important that these features are retained when flow-reactor volume is increased. If this is not the case then the same issues arise as observed in batch when a process fails to scale either from a changing product-quality or safety perspective.

## Experimental

**Materials.** In all cases, materials were used as received from Acros Organics, with reaction solvents purchased as “Extra Dry” and stored over molecular sieves and analytical grade solvents purchased for use in aqueous extractions.

**Instrumentation.** Unless otherwise stated, nuclear magnetic resonance (NMR) spectra were obtained at room temperature from solutions in deuterated chloroform (CDCl_3_ 0.01% TMS) by means of a Jeol GX400 spectrometer; in the case of known compounds, all spectra obtained were consistent with the literature. The following abbreviations are used to report NMR spectroscopic data; s = singlet, d = doublet, t = triplet, br s = broad singlet, q = quartet, dd = double doublet, dt = doublet of triplets, m = multiplet and C_0_ = quaternary carbon. Analysis of samples by gas chromatography-flame ionisation detection (GC-FID) were performed on a Varian GC (430) with a CP-Sil 8 (30 m) column (Phenomenex, UK) and ultrahigh purity helium (99.9999%, Energas, UK) as the carrier gas. Reaction products were analysed by the following method; injector temperature 200 °C, carrier-gas flow rate 1.60 mL·min^−1^, oven temperature 50 °C for 0.1 min then ramped to 300 °C at 60 °C·min^−1^ and held at 300 °C for 1.0 min (Table 3). Mass spectrometry data was obtained by means of a Shimadzu QP5050A instrument with an EI ionisation source.

**Table 3:** Summary of the retention times obtained for the key starting materials and products employed herein.

Analyte	Retention time (min)	Purity (%)

3,4-Dichloronitrobenzene (**4**)	3.20	99.0
4-Methoxyphenol (**5**)	3.36	98.0
4-Nitrophenol (**8**)	3.47	99.0
4-Cyanophenol (**9**)	3.22	99.0
4-Bromophenol (**10**)	2.92	97.0
4-Fluorophenol (**11**)	2.23	99.0
2-Chloro-1-(4-methoxyphenoxy)-4-nitrobenzene (**7**)	5.10	99.99^a^
2-Chloro-1-(4-nitrophenoxy)-4-nitrobenzene (**12**)	5.50	99.98^a^
2-Chloro-1-(4-cyanophenoxy)-4-nitrobenzene (**13**)	6.81	99.99^a^
2-Chloro-1-(4-bromophenoxy)-4-nitrobenzene (**14**)	5.07	99.99^a^
2-Chloro-1-(4-fluorophenoxy)-4-nitrobenzene (**15**)	4.37	99.98^a^

^a^As determined by GC-FID analysis.

**Figure 11:**
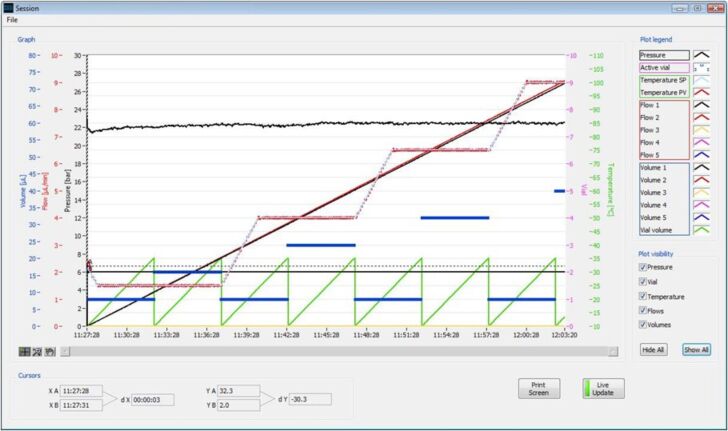
Graphical representation of an automated flow reaction for equilibration and screening of reactor temperature effects.

**Microreactor setup:** Microreactions were performed in the Labtrix^®^ S1 (Chemtrix BV, NL), illustrated in Figure 2, fitted with a glass microreactor (3223, reactor volume = 10 µL) containing an SOR-2 static micromixer. Reactant solutions were introduced into the reactor through three 1 mL gas-tight syringes (SGE, UK) capable of delivering three solutions at flow rates between 0.1 and 100 μL·min^−1^. The system was maintained at 25 bar of back pressure by means of a preset ultralow dead-volume back-pressure regulator (Upchurch Scientific, USA), in order to prevent boiling of the reactants and solvent system when temperatures above the atmospheric boiling point were employed. The system was controlled through the Labtrix^®^ S1 software, which enables control of reactant flow rate (total flow rate ≤80 µL·min^−1^, reactant residence time (7.5 s to 50 min (for a 10 µL reactor)), reactor temperature (−15 to 195 °C), equilibration time and sample collection into one of twenty-nine 2 mL sample vials. The software also archives system parameters such as the set and actual temperature, system pressure, reactor type, and flow rates programmed, along with the sample collection time and vessel, for review both during and after the experiment (Figure 11).

**General procedure for temperature and base screening.** By using the Labtrix^®^ S1, fitted with a glass microreactor (3223, reactor volume = 10 µL) and a back-pressure regulator set to 25 bar, thermostatted to 25 °C, a solution of 3,4-dichloronitrobenzene (**4**) and phenol derivative (1.3 M respectively) was pumped into the reactor from inlet 1, a solution of base (1.00 M or 1.95 M) was introduced from inlet 2 and the solvent system under investigation was introduced as a diluent from inlet 3. After the system volume had passed through the reactor three times the reaction was at steady state and a sample then collected and analysed offline by GC-FID (Table 3). The reactor temperature was then increased by 25 °C and the system allowed to equilibrate before a sample was taken for analysis in order to quantify the proportion of diaryl ether synthesised; this procedure was repeated up to the *T*_max_ (195 °C) of the system.

**General procedure for diaryl ether synthesis with DBU** (**6**)**.** By using the Labtrix^®^ S1, fitted with a glass microreactor (3223, reactor volume = 10 µL) and a back-pressure regulator set to 25 bar, a solution of 3,4-dichloronitrobenzene (**4**) and phenol derivative (1.3 M respectively) was pumped into the reactor from inlet 1 and a solution of 1,8-diazabicyclo[5.4.0]undec-7-ene (**6**, 1.95 M) was introduced from inlet 2. After a reactant residence time of 1 to 10 min (Table 2), a 500 µL aliquot of the reaction product was collected in a round-bottomed flask and concentrated in vacuo to remove the reaction solvent prior to aqueous extraction. The organic residue was dissolved in DCM (25 mL) and then washed with an aqueous solution of saturated ammonium chloride (3 × 25 mL) to remove the organic base. The organic layer was then dried with MgSO_4_, filtered under suction and the filtrate concentrated in vacuo to afford the target diaryl ether. The reaction product was then analysed by mass spectrometry and ^1^H/^13^C NMR spectroscopy in order to characterise the ether and determine the product purity.

**2-Chloro-1-(4-methoxyphenoxy)-4-nitrobenzene** (**7**)**.** A solution of DCNB (**4**) and 4-methoxyphenol (**5**, 0.2496 g and 0.1614 g·mL^−1^, 1.3 M) in MeCN was introduced into the microreactor at a flow rate of 5 µL·min^−1^ and a solution of DBU (**6**, 0.2968 mL·mL^−1^, 1.95 M) in MeCN was introduced at a flow rate of 5 µL·min^−1^. The microreactor was heated to 195 °C (in 25 °C stages) and, after an equilibration time of 3 min, 500 µL of reaction product was collected into a round-bottomed flask (10 mL) and concentrated in vacuo to remove the reaction solvent prior to aqueous extraction. The organic residue was dissolved in DCM (25 mL) and then washed with an aqueous solution of saturated ammonium chloride (3 × 25 mL) to remove the organic base. The organic layer was then dried with MgSO_4_, filtered under suction and the filtrate concentrated in vacuo to afford the target diaryl ether, affording 2-chloro-1-(4-methoxyphenoxy)-4-nitrobenzene (**7**) as a pale yellow solid (90.4 mg, 99.7%); ^1^H NMR (400 MHz, CDCl_3_) δ 3.87 (3H, s, OCH_3_), 6.80 (1H, dd, *J* = 3.0 and 9.2, 1 × ArH), 6.96 (2H, d, *J* = 9.0, 2 × 1 ArH), 7.04 (2H, d, *J* = 9.0, 2 × ArH), 8.01 (1H, dd, *J* = 3.0 and 9.2, 1 × ArH) and 8.35 (1H, d, *J* = 3.0, 1 × ArH); ^13^C NMR (100 MHz, CDCl_3_) δ 55.6 (OCH_3_), 115.3 (2 × CH), 115.5 (CH), 121.6 (2 × CH), 123.5 (CH), 123.8 (C_0_), 126.4 (CH), 142.1 (C_0_NO_2_), 147.4 (C_0_), 157.3 (C_0_) and 159.8 (C_0_); *m*/*z* (EI) 280 (M^+^ + 1, 25%), 279 (100), 264 (20), 233 (10), 198 (7), 183 (5), 123 (5), 108 (2) and 76 (5). The spectroscopic data obtained were consistent with those reported in the literature [15].

**2-Chloro-1-(4-methoxyphenoxy)-4-nitrobenzene** (**7**)**.** A solution of DCNB (**4**) and 4-methoxyphenol (**5**, 0.2496 g and 0.1614 g·mL^−1^, 1.3 M) in MeCN was introduced to the microreactor at a flow rate of 7 µL·min^−1^ and a solution of K_2_CO_3_ (4.5 M) in MeCN was introduced at a flow rate of 3 µL·min^−1^. The microreactor was heated to 195 °C (in 25 °C stages) and, after an equilibration time of 3 min, 500 µL of reaction product was collected in a round-bottomed flask (10 mL) and concentrated in vacuo to remove the reaction solvent prior to aqueous extraction. The organic residue was dissolved in DCM (25 mL) and then washed with an aqueous solution of saturated ammonium chloride (3 × 25 mL) to remove the organic base. The organic layer was then dried with MgSO_4_, filtered under suction and the filtrate concentrated in vacuo to afford the target diaryl ether, affording 2-chloro-1-(4-methoxyphenoxy)-4-nitrobenzene (**7**) as a pale yellow solid (152.0 mg, 99.8%); spectroscopic data obtained were consistent with those reported above and in the literature [15].

**2-Chloro-1-(4-nitrophenoxy)-4-nitrobenzene** (**12**)**:** A solution of DCNB (**4**) and 4-nitrophenol (**8**, 0.2496 g and 0.1899 g·mL^−1^, 1.3 M) in MeCN was introduced to the microreactor at a flow rate of 1 µL·min^−1^ and a solution of DBU (**6**, 0.2968 mL·mL^−1^, 1.95 M) in MeCN was introduced at a flow rate of 1 µL·min^−1^. The microreactor was heated to 195 °C (in 25 °C stages) and, after an equilibration time of 15 min, 500 µL of reaction product was collected in a round-bottomed flask (10 mL) and concentrated in vacuo to remove the reaction solvent prior to aqueous extraction. The organic residue was dissolved in DCM (25 mL) and then washed with an aqueous solution of saturated ammonium chloride (3 × 25 mL) to remove the organic base. The organic layer was then dried with MgSO_4_, filtered under suction and the filtrate concentrated in vacuo to afford the target diaryl ether, affording 2-chloro-1-(4-nitrophenoxy)-4-nitrobenzene (**12**) as a yellow solid (95.4 mg, 99.8%); ^1^H NMR (400 MHz, CDCl_3_) δ 7.12 (2H, d, *J* = 9.1, 2 × ArH), 7.21 (1H, d, *J* = 9.0, 1 × ArH), 8.21 (1H, dd, *J* = 2.8 and 9.0, 1 × ArH), 8.30 (2H, d, *J* = 9.1, 2 × ArH) and 8.42 (1H, d, *J* = 2.8, 1 × ArH); ^13^C NMR (100 MHz, CDCl_3_) δ 118.2 (2 × CH), 120.7 (CH), 123.9 (CH), 126.2 (2 × CH), 126.8 (CH), 127.0 (C_0_Cl), 144.1 (C_0_NO_2_), 144.5 (C_0_NO_2_), 160.4 (C_0_O) and 160.6 (C_0_O); *m*/*z* (EI) 297 (M^+^ + 1, 25%) 296 (65), 295 (55), 294 (50), 282 (45), 267 (25), 265 (45), 264 (100), 251 (7), 249 (20), 167 (10), 91 (15) and 76 (10). The spectroscopic data obtained were consistent with those reported in the literature [15].

**2-Chloro-1-(4-cyanophenoxy)-4-nitrobenzene** (**13**)**:** A solution of DCNB (**4**) and 4-cyanophenol (**9**) (0.2496 g and 0.1548 g·mL^−1^, 1.3 M) in MeCN was introduced to the microreactor at a flow rate of 0.5 µL·min^−1^ and a solution of DBU (**6**, 0.2968 mL·mL^−1^, 1.95 M) in MeCN was introduced at a flow rate of 0.5 µL·min^−1^. The microreactor was heated to 195 °C (in 25 °C stages) and, after an equilibration time of 30 min, 500 µL of reaction product was collected in a round-bottomed flask (10 mL) and concentrated in vacuo to remove the reaction solvent prior to aqueous extraction. The organic residue was dissolved in DCM (25 mL) and then washed with an aqueous solution of saturated ammonium chloride (3 × 25 mL) to remove the organic base. The organic layer was then dried with MgSO_4_, filtered under suction and the filtrate concentrated in vacuo to afford the target diaryl ether, affording 2-chloro-1-(4-cyanophenoxy)-4-nitrobenzene (**13**) as a pale yellow solid (88.8 mg, 99.72%); ^1^H NMR (400 MHz, CDCl_3_) δ 7.10 (2H, d, *J* = 8.8, 2 × ArH), 7.14 (1H, d, *J* = 9.1, 1 × ArH), 7.27 (2H, d, *J* = 8.8, 2 × ArH), 8.16 (1H, dd, *J* = 2.8 and 9.1, 1 × ArH) and 8.42 (1H, d, *J* = 2.8, 1 × ArH); ^13^C NMR (100 MHz, CDCl_3_) δ 108.4 (C_0_CN), 118.1 (CN), 119.0 (2 × CH), 120.3 (CH), 123.8 (CH), 126.8 (C_0_Cl), 126.9 (CH), 134.6 (2 × CH), 144.4 (C_0_NO_2_), 156.3 (C_0_O) and 158.8 (C_0_O); *m*/*z* (EI) 277 (M^+^ + 1, 15%), 276 (30), 275 (17), 274 (100), 267 (10), 245 (15), 243 (12), 198 (35), 181 (20), 167 (5), 92 (10) and 76 (15). The spectroscopic data obtained were consistent with those reported in the literature [15].

**2-Chloro-1-(4-bromophenoxy)-4-nitrobenzene** (**14**)**:** A solution of DCNB (**4**) and 4-bromophenol (**10**, 0.2496 g and 0.2249 g·mL^−1^, 1.3 M) in MeCN was introduced to the microreactor at a flow rate of 5 µL·min^−1^, a solution of DBU (**6**, 0.2968 mL·mL^−1^, 1.95 M) in MeCN was introduced at a flow rate of 5 µL·min^−1^ and acetone was introduced at a flow rate of 10 µL·min^−1^ (to prevent crystallisation of the product in the outlet tube). The microreactor was heated to 195 °C (in 25 °C stages) and, after an equilibration time of 3 min, 500 µL of reaction product was collected into a round-bottomed flask (10 mL) and concentrated in vacuo to remove the reaction solvent prior to aqueous extraction. The organic residue was dissolved in DCM (25 mL) and then washed with an aqueous solution of saturated ammonium chloride (3 × 25 mL) to remove the organic base. The organic layer was then dried with MgSO_4_, filtered under suction and the filtrate concentrated in vacuo to afford the target diaryl ether, affording 2-chloro-1-(4-bromophenoxy)-4-nitrobenzene (**14**) as a yellow solid (106.1 mg, 99.86%); ^1^H NMR (400 MHz, CDCl_3_) δ 6.90 (1H, d, *J* = 9.1, 1 × ArH), 6.97 (2H, d, *J* = 6.9, 2 × ArH), 7.54 (1H, d, *J* = 6.9, 2 × ArH), 8.06 (1H, dd, *J* = 2.8 and 9.1, 1 × ArH) and 8.37 (1H, d, *J* = 2.8, 1 × ArH); ^13^C NMR (100 MHz, CDCl_3_) δ 117.2 (CH), 118.4 (C_0_Br), 121.6 (2 × CH), 123.6 (CH), 125.0 (C_0_Cl), 126.6 (CH), 133.4 (2 × CH), 143.0 (C_0_NO_2_), 153.7 (C_0_O) and 158.3 (C_0_O); *m*/*z* (EI) 330 (M^+^ + 1, 4%), 329 (100), 328 (3), 327 (75), 313 (3), 297 (5), 283 (5), 203 (20), 171 (10), 139 (25), 108 (5) and 76 (5). The spectroscopic data obtained were consistent with those reported in the literature [15].

**2-Chloro-1-(4-fluorophenoxy)-4-nitrobenzene** (**15**)**:** A solution of DCNB (**4**) and 4-fluorophenol (**11**, 0.2496 g and 0.1457 g·mL^−1^, 1.3 M) in MeCN was introduced to the microreactor at a flow rate of 2.5 µL·min^−1^, a solution of DBU (**6**, 0.2968 mL·mL^−1^, 1.95 M) in MeCN was introduced at a flow rate of 2.5 µL·min^−1^ and acetone was introduced at a flow rate of 10 µL·min^−1^ (to prevent product crystallisation in the outlet tube). The microreactor was heated to 195 °C (in 25 °C stages) and, after an equilibration time of 8 min, 500 µL of reaction product was collected into a round-bottomed flask (10 mL) and concentrated in vacuo to remove the reaction solvent prior to aqueous extraction. The organic residue was dissolved in DCM (25 mL) and then washed with an aqueous solution of saturated ammonium chloride (3 × 25 mL) to remove the organic base. The organic layer was then dried with MgSO_4_, filtered under suction and the filtrate concentrated in vacuo to afford the target diaryl ether affording 2-chloro-1-(4-fluorophenoxy)-4-nitrobenzene (**15**) as a cream-coloured solid (86.6 mg, 99.79%); ^1^H NMR (400 MHz, CDCl_3_) δ 6.83 (1H, d, *J* = 9.1, 1 × ArH), 7.08–7.15 (4H, m, 4 × ArH), 8.06 (1H, dd, *J* = 2.8 and 9.1, 1 × ArH) and 8.38 (1H, d, *J* = 2.8, 1 × ArH); ^13^C NMR (100 MHz, CDCl_3_) δ 116.3 (CH), 117.1 (2 × CH, d, *J* = 23.7), 121.8 (2 × CH, d, *J* = 8.4), 123.6 (CH), 124.5 (C_0_Cl), 126.6 (CH), 142.7 (C_0_NO_2_), 150.2 (C_0_O, d, *J* = 3.0), 160.1 (C_0_F, d, *J* = 243.9) and 161.3 (C_0_O); *m*/*z* (EI) 269 (M^+^ + 1, 35%), 268 (19), 267 (100), 249 (10), 222 (15), 186 (40), 157 (30), 139 (10), 112 (5), 107 (5) and 76 (7). The spectroscopic data obtained were consistent with those reported in the literature [15].

## References

[1] Frlan R, Kikelj D (2006). Synthesis.

[2] Boger D L, Yohannes D (1989). J Org Chem.

[3] Gottsegen Á, Nógrádi M, Vermes B, Kajtár-peredy M, Bihátsi-karsai É (1988). Tetrahedron Lett.

[4] Cirla A, Mann J (2003). Nat Prod Rep.

[5] Johnson W O, Kollman G E, Swithenbank C, Yih R Y (1978). J Agric Food Chem.

[6] Chan D M T, Monaco K L, Wang R-P, Winters M P (1998). Tetrahedron Lett.

[7] Jung M E, Jachiet D, Khan S I, Kim C (1995). Tetrahedron Lett.

[8] Burgos C H, Barder T E, Huang X, Buchwald S L (2006). Angew Chem, Int Ed.

[9] Kulagowski J J, Rees C W (1980). Synthesis.

[10] Sawyer J S, Schmittling E A, Palkowitz J A, Smith W J (1998). J Org Chem.

[11] Zhao J K, Wang Y G (2003). Chin Chem Lett.

[12] Jung N, Bräse S (2009). J Comb Chem.

[13] Ueno M, Yonemoto M, Hashimoto M, Wheatley A E H, Naka H, Kondo Y (2007). Chem Commun.

[14] Lee J-K, Fuchter M J, Williamson R M, Leeke G A, Bush E J, McConvey I F, Saubern S, Ryan J H, Holmes A B (2008). Chem Commun.

[15] Marafie J A, Moseley J D (2010). Org Biomol Chem.

[16] Moseley J D, Woodman E K (2009). Energy Fuels.

[17] Moseley J D, Kappe C O (2011). Green Chem.

[18] Iannelli M, Bergamelli F, Kormos C M, Paravisi S, Leadbeater N E (2009). Org Process Res Dev.

[19] Razzaq T, Kappe C O (2008). ChemSusChem.

[20] Dressen M H C L, van de Kruijs B H P, Meuldijk J, Vekemans J A J M, Hulshof L A (2010). Org Process Res Dev.

[21] Damm M, Glasnov T, Kappe C O (2010). Org Process Res Dev.

[22] Wienhöfer I C, Studer A, Rahman M T, Fukuyama T, Ryu I (2009). Org Lett.

[R23] 23Wiles, C.; Watts, P. In *Micro reactors in organic synthesis*; CRC-Press, 2011.

[24] Wiles C, Watts P (2011). Chem Commun.

[R25] 25Luis, S. V.; Garcia-Verdugo, E. In *Chemical reactions and processes under flow conditions;* Royal Society of Chemistry, 2010.

[26] Fu X, Liu S, Ruan X, Yang H (2006). Sens Actuators, B.

[R27] 27DBU (**6**) = £51.80/100g compared with £7.50/100 g for K_2_CO_3_ (www.sigmaaldrich.com (21/05/11)).

